# Computed tomography coronary angiography in patients without known coronary artery disease can demonstrate possible non-cardiovascular causes of non-acute retrosternal chest pain

**DOI:** 10.1007/s13244-018-0654-x

**Published:** 2018-10-01

**Authors:** Silvia Tresoldi, Anna Ravelli, Sara Sbaraini, Claudia Khouri Chalouhi, Francesco Secchi, Gianpaolo Cornalba, Gianpaolo Carrafiello, Francesco Sardanelli

**Affiliations:** 1 Unit of Diagnostic and Interventional Radiology, Department of Diagnostic Services, ASST Santi Paolo e Carlo, via A. di Rudinì 8, 20142 Milan, Italy; 2 Radiology, Ospedale dei bambini “Vittore Buzzi”, via Castelvetro 32, 20154 Milan, Italy; 3 Postgraduation School in Radiodiagnostics, Università degli Studi di Milano, via Festa del Perdono 7, 20122 Milan, Italy; 4 Radiology, Grande Ospedale Metropolitano Niguarda, Piazza dell’Ospedale Maggiore 3, 20162 Milan, Italy; 5 Unit of Radiology, Research Hospital Policlinico San Donato, via Morandi 30, San Donato Milanese, 20097 Milan, Italy; 6 Department of Sciences for Health, Università degli Studi di Milano, via A. di Rudinì 8, 20142 Milan, Italy; 7 Department of Biomedical Sciences for Health, Università degli Studi di Milano, via Morandi 30, San Donato Milanese, 20097 Milan, Italy

**Keywords:** Chest pain, Computed tomography coronary angiography, Coronary artery disease, Differential diagnosis

## Abstract

**Objectives:**

To assess the computed tomography coronary angiography (CTCA) accuracy for demonstrating possible non-cardiovascular causes of non-acute retrosternal chest pain in patients without known coronary artery disease (CAD) and to correlate CTCA results with the patient management and relief from pain.

**Methods:**

This prospective observational study was approved by the ethical committee. Consecutive patients suffering non-acute chest pain who underwent CTCA and with not known CAD were enrolled and classified as having coronary diseases (CD) or extracardiac diseases (ECD). Association between age, sex, body mass index (BMI), cardiovascular risk factors, and type of chest pain with CD or ECD was estimated. Correlation between BMI classes and each risk factor was also calculated.

**Results:**

A total of 106 patients (60 males; age 62 ± 14 years [mean ± standard deviation]; mean BMI 27) were enrolled. Hypertension was found in 71/106 (67%); smoking was significantly more frequent among males (*p* = 0.003) and hypercholesterolemia among females (*p* = 0.017); hypertension and hypercholesterolemia significantly correlated with age, and hypertension also with BMI. Pain was atypical in 70/106 (66%) patients. The kind of pain did not correlate with disease or gender. CTCA showed possible causes of chest pain in 69/106 (65%) patients; 32/69 (47%) having only CD, 23/69 (33%) only ECD, and 14/69 (20%) both CD and ECD. Prevalence was: hiatal hernia 35/106 (33%); significant CAD 24/106 (23%); myocardial bridging 22/106 (21%). At follow-up of 94/106 (89%) patients, 71/94 (76%) were pain-free, 14/17 (82%) significant CAD had been treated, and only one patient with non-significant CAD was treated after CTCA.

**Conclusion:**

CTCA suggested possible causes of non-acute pain in 65% of patients.

**Main messages:**

• *CTCA can either rule in or rule out possible causes of chest pain alternative to CAD.*

• *Clinically relevant findings were detected in 65% of patients with non-acute chest pain.*

• *Non-cardiovascular diseases potentially explained symptoms in 35% of patients.*

## Introduction

The differential diagnosis of retrosternal chest pain can be difficult [1, 2]. Angina-like retrosternal chest pain can arise either from cardiovascular or from non-cardiovascular causes, such as hiatal hernia and esophageal disease [1–3]. The underlying problem is the common sensory innervation of heart, pleura, aorta, and esophagus by fibers from the same spinal segments [2–4]. Moreover, patient history does not have a high predictive value for the origin of chest pain [2].

Most patients with retrosternal chest pain consult a cardiologist. This often results in a late referral to other specialists, after a cardiac origin of the symptoms had been definitely excluded, with persistent patient discomfort for months after the first painful episode [2].

In recent years, the use of computed tomography (CT) in the evaluation of coronary artery disease (CAD) has spread [5–9]. Current data show that, in low-to-intermediate risk patients, ≥ 64-slice CT coronary angiography (CTCA) should be considered the method for ruling out coronary origin of chest pain [10–13]. This allows many patients with non-significant CAD at CTCA to avoid conventional coronary angiography. Of note, CTCA is performed as a contrast-enhanced chest CT with an only slightly reduced field of view, evaluating simultaneously coronary arteries, the whole heart, and many other thoracic structures.

The first aim of our study was to assess, in a population of patients without previous CAD history, referred to CTCA for retrosternal chest pain, the prevalence of both coronary/cardiovascular disease and non-cardiovascular diseases, and correlate them with the kind of chest pain. The second aim was to perform a clinical follow-up in order to evaluate the correlations between CTCA results, patient management, and potential relief from chest pain.

## Materials and methods

### Study population

This prospective observational study was approved by the ethical committee and written informed consent was obtained from all patients. All consecutive patients who arrived in the Radiology Department of our hospital, between July 2010 and September 2014, to perform a CTCA for many clinical questions (e.g., suspect CAD in patients with new evidence of dilated cardiomyopathy, suspect CAD in patients with discordance between two different provocative tests, suspect CAD in patients with discordance between symptoms and provocative test, suspect CAD in patients with symptoms and many risk factors), and affected by retrosternal chest pain, were prospectively included. Patients with acute chest pain, directly referred to the Emergency Department, were excluded, as well as patients with known CAD. For each patient, a form with data about age, sex, body mass index (BMI), cardiovascular risk factors, and type of chest pain—*typical* (three criteria of NICE 2016 classification) or *no-cardiac origin*/*atypical* (one or two criteria of NICE 2016 classification)—was recorded [3, 14].

### CTCA protocol

In all patients with a resting heart rate > 65 bpm and no contraindications, metoprolol (50–100 mg) was orally administered about 1 h before the examination to achieve a target heart rate ≤ 65 bpm. Administration of sublingual nitroglycerin (0.30 mg) was used to enhance coronary vasodilatation at the time of imaging. CTCA examinations were performed using a LightSpeed VCT (GE Healthcare, Milwaukee, WI, USA), with retrospective ECG gating, end-diastolic (60–80% of the R-R cardiac cycle) craniocaudal reconstruction (carina to diaphragm), detector configuration 64 × 0.625 mm, acquisition thickness 0.625 mm, acquisition interval 0.625 mm, tube rotation time 0.4 s, tube voltage 80–120 kV (based on patient body weight), ECG-dependent tube current modulation settled to a reference of 250–700 mAs in the 60–80% RR interval, and of 250mAs for the rest of the cardiac circle (with an average effective radiation dose ≤ 15 mSv), acquisition field of view (FOV) 25 × 25 cm, standard filter kernel, and mediastinal CT window.

All patients received a bolus of 85 mL of contrast material (Iomeron 400 mgI/mL, Bracco Imaging SpA, Milan, Italy) through an antecubital vein at 5 mL/s, followed by 50 mL of saline solution at the same rate. The scan delay was determined using the bolus tracking technique, positioning a region of interest with a triggering threshold preset at 150 HU on the descending aorta at the level of the four-chamber view. Images were reconstructed with a thickness 0.625 mm and an interval of 0.4 mm, as well as contiguous 2.5 mm, with an enlarged FOV (32 cm × 32 cm) and lung and mediastinal window for the evaluation of lung parenchyma and other structures included in the acquisition volume.

### Image analysis

**Fig. 1:**
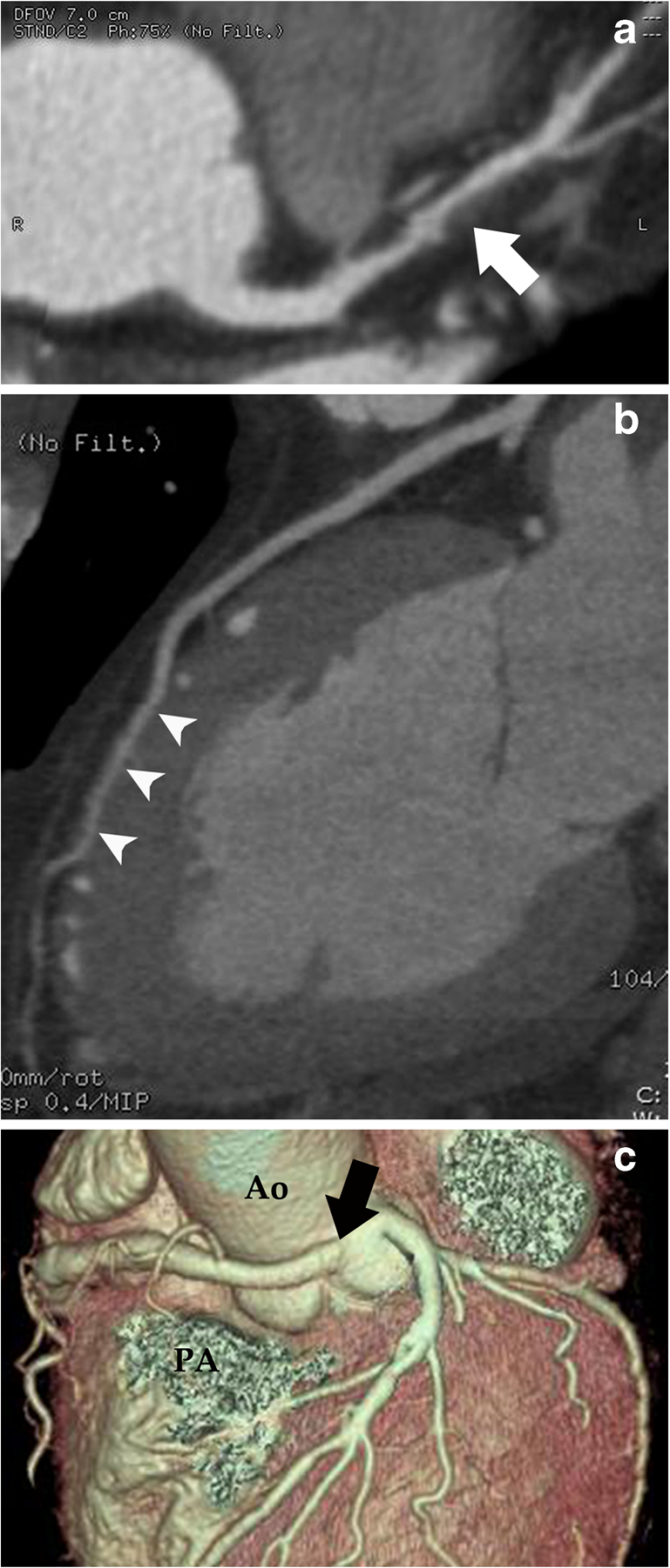
Different kinds of computed tomography coronary angiography (CTCA) images showing examples of coronary diseases. **a** Transverse curved planar reformatted (CPR) image showing a non-calcified plaque in the mid-segment of the left anterior descending coronary artery (*arrow*), causing a significant (> 50%) stenosis. **b** CPR image along the left ventricle vertical long axis showing a 3-cm-long myocardial bridging (*arrowheads*) of the mid-distal third of the left anterior descending coronary artery. **c** Three-dimensional volume rendering image showing the origin from the left Valsalva sinus of the right coronary artery (*arrow*), which reaches the right atrium-ventricular path, coursing between the aorta root (*Ao*) and the pulmonary artery root (cut – *PA*) (malignant course)

**Fig. 2:**
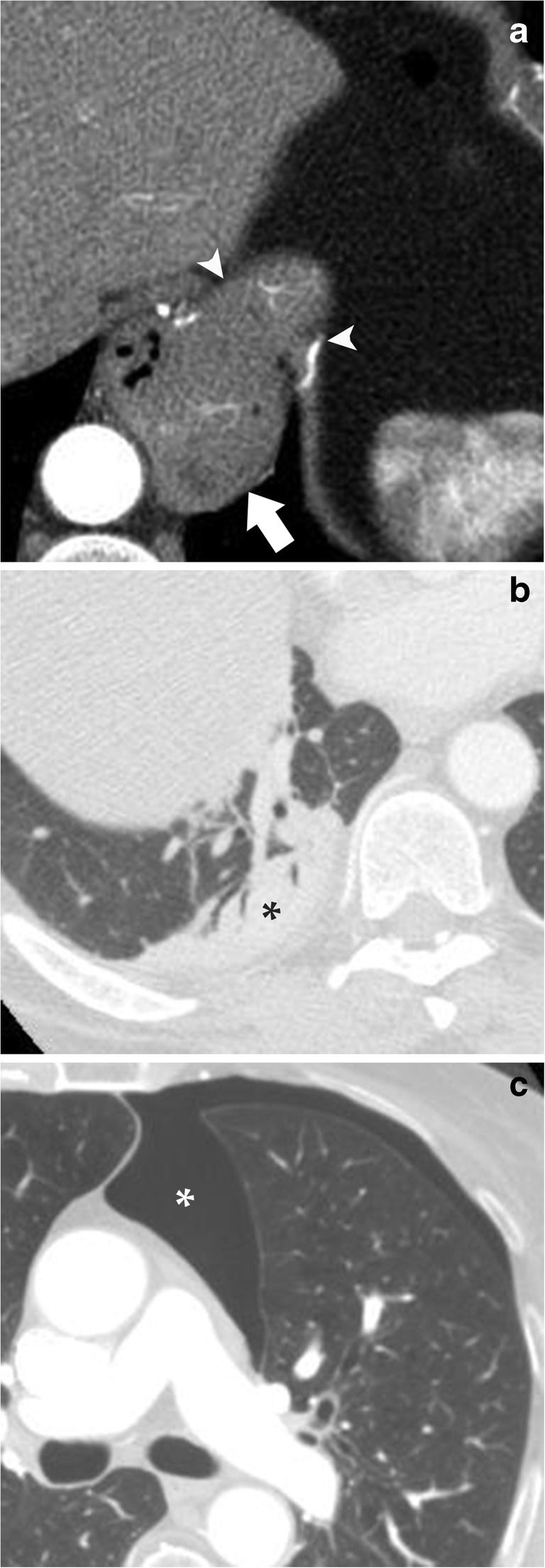
CT pure axial images showing examples of extracardiac diseases. **a** Axial image at the supra-diaphragmatic level showing a large hiatal hernia (*arrow*) extending into the thoracic cavity through the esophageal hiatus of the diaphragm (*arrowheads*). **b** Axial image with lung window, at the heart level, showing pulmonary consolidation (*asterisk*) with air bronchogram in the posterior-basal segment of the right lung. **c** Axial image with lung window, at the pulmonary artery bifurcation level, showing a significant anterior mainly para-mediastinal pneumothorax (*asterisk*) of the left lung

CTCA data sets were analyzed using a dedicated workstation (Advantage Workstation 4.4, GE Healthcare, Milwaukee, WI, USA). Evaluation of coronary arteries was performed by a certified radiologist (S.T.) with 8 years of experience in cardiac CT on axial, multi-planar reformatted (MPR) images, and curved planar reformatted (CPR) images along the centerline of each vessel; if necessary, maximum intensity projection (MIP) and volume rendering (VR) images were used. In the presence of calcified, non-calcified, or mixed plaques in at least one coronary segment (defined following the 17-segment modified AHA classification), coronary atherosclerosis was diagnosed and classified following the grading system recommended by the Society of Cardiovascular Computed Tomography (normal, absence of plaque/no luminal stenosis; minimal, < 25% stenosis; mild, 25–49% stenosis; moderate, 50–69% stenosis; severe, 70–99% stenosis) [15]. We defined “significant disease” as the presence of moderate or severe stenosis (Fig. 1a) and “non-significant disease” as the presence of minimal or mild stenosis. No functional data (e.g., non-invasive fractional flow reserve) were considered. The presence of either other coronary non-atherosclerotic diseases (i.e., myocardial bridging, Fig. 1b, or malignant coronary anomalies, Fig. 1c) or extracardiovascular findings which were possible causes of chest pain (Fig. 2) was assessed on the same images set. We defined any tract of coronary arteries or their major branches that courses beneath the myocardium as myocardial bridging and coronaries originating from the opposite Valsalva sinus and crossing over to their regular peripheral locations with an interarterial course between the pulmonary artery and aorta as malignant coronary anomalies.

### Follow-up

Follow-up was conducted either by means of telephone interviews or by reviewing the hospital medical records, such as hospital discharge letters, provocative tests, or conventional coronary angiography results, with a wide follow-up time range, mainly due to the duration of the study and different follow-up modalities. For each patient, the definitive diagnosis and treatment choice were decided by the referring cardiologist or general practitioner. Correlations between CTCA results, patient management, and relief from chest pain were analyzed.

### Statistical analysis

Continuous variables are expressed as mean ± standard deviation (SD), whereas categorical variables are indicated as frequencies or percentages. Association between gender, age, and risk factors, including BMI, was estimated with the χ^2^ or Fisher’s exact test. Correlation between BMI and risk factors was estimated with Spearman’s correlation. Association between type of chest pain and presence of cardiovascular alterations was estimated using the χ^2^ or Fisher’s exact test. All analyses were performed using SPSS v.21 (SPSS Inc., Chicago, IL) and *p*-values < 0.05 were considered significant.

## Results

### Study population

The study population included 106 patients (60 males, 46 females), aged 62 ± 14 years (mean ± SD; range 21–93 years). Indications for CTCA were: discordance between symptoms and provocative test (*n* = 48), new evidence of dilated cardiomyopathy (*n* = 16), symptoms and many risk factors (*n* = 13), discordance between two different provocative tests (*n* = 10), recent diagnosis of acute coronary syndrome (*n* = 10), and suspected coronary anomaly (*n* = 9).

Most of the study population was overweight (BMI 27 ± 6 [mean ± SD]; range 17–57), with a significant difference in weight between males (BMI 28 ± 5; range 19–49) and females (BMI 26 ± 7; range 17–56) (*p* = 0.043). Hypertension was the most common risk factor (71/106, 67%), while diabetes was the least frequent risk factor (17/106, 16%). Smoking habit was significantly more common among males (34/60, 57%) than among females (13/46, 28%) (*p* = 0.003; Fisher’s exact test). More females (28/46, 61%) than males (23/60, 38%) had hypercholesterolemia (*p* = 0.017). The distribution of hypertension and hypercholesterolemia in the study population was significantly correlated to patient age (r_s_ = 0.237; *p* = 0.014 and r_s_ = 0.261; *p* = 0.007, respectively). Moreover, a significant correlation was found between hypertension and BMI classes (r_s_ = 0.315; *p* = 0.001).

Thirty-four percent (36/106) of patients had typical and 66% (70/106) atypical chest pain, with no significant differences between genders (*p* = 0.357, Fisher’s exact test).

### Image analysis

All of the CT examinations were diagnostic, including the six cases of patients who had irregular heart rhythm during images acquisition: 65–70 bpm in the presence of 1–2 ectopic beats (*n* = 3); atrial fibrillation (*n* = 2); multiple ventricular ectopic beats (*n* = 1). The heart rate was 62 ± 10 bpm (mean ± SD; range: 40–94 bpm). Thirty-five percent (37/106) of patients did not present any pathological findings at the CT scan, 30% (32/106) were diagnosed with only coronary disease (Fig. 1), and 22% (23/106) with only non-cardiovascular disease (Fig. 2). Thirteen percent (14/106) of patients presented both a coronary and ≥ 1 non-cardiovascular disease: myocardial bridging and hiatal hernia (8/106); significant CAD and hiatal hernia (3/106); significant CAD, myocardial bridging, and hiatal hernia (2/106); malignant coronary anomaly and hiatal hernia (1/106). Table 1 presents all of the CT findings in relation to chest pain. The main possible causes of chest pain were hiatal hernia (33%, 35/106), significant CAD (23%, 24/106), and myocardial coronary bridging (22%, 22/106).

**Table 1:** Association between computed tomography (CT) results and chest pain among the 106 patients of the study population

CT results		Chest pain		
		Atypical, *n* (%)	Typical, *n* (%)	Total, *n* (%)
Nothing		27 (25)	10 (9)	37 (35)
Coronary diseases (CD) only	Significant CAD	12 (11)	7 (7)	19 (18)
	Myocardial bridging	7 (7)	5 (5)	12 (11)
	Malignant coronary anomaly	0 (0)	1 (1)	1 (1)
	Total	19 (18)	13 (12)	32 (30)
CD + ECD	Myocardial bridging + hiatal hernia	6 (6)	2 (2)	8 (8)
	Significant CAD + hiatal hernia	2 (2)	1 (1)	3 (3)
	Significant CAD + myocardial bridging + hiatal hernia	1 (1)	1 (1)	2 (2)
	Malignant coronary anomaly + hiatal hernia	1 (1)	0 (0)	1 (1)
	Total	10 (9)	4 (4)	14 (13)
Extracardiac diseases (ECD) only	Hiatal hernia	12 (11)	9 (8)	21 (20)
	Pneumothorax	1 (1)	0 (0)	1 (1)
	Pneumonia + cholecystitis	1 (1)	0 (0)	1 (1)
	Total	14 (13)	9 (8)	23 (22)
Total		70 (66)	36 (34)	106 (100)

Almost 80% of patients (82/106) presented with normal coronary arteries or minimal/mild CAD, the majority of whom presented with atypical pain (55/82; 67%). Among patients with typical pain (36/106), 25% (9/36) had significant CAD. No significant association was demonstrated between the kind of chest pain (typical versus atypical) and any disease detected at CT (coronary *p* = 0.955; non-cardiovascular *p* = 0.590).

### Follow-up

Follow-up information was available for 94/106 patients (89%), with a median follow-up time of 33 months (range 7–57). In Table 2, the association between CT results and the patient management among the 94 patients with available follow-up is summarized. Seventy-five percent (71/94) of these patients no longer experienced chest pain. Considering these 94 patients, the results of follow-up were: free of any disease, 36% (34/94); evidence of cardiovascular disease, 40% (38/94); evidence of non-cardiovascular disease, 38% (36/94). Among patients with patent coronary arteries at CTCA (34/94), seven were diagnosed with myocarditis and promptly treated at the time of diagnosis with pain relief, four received cardiologic therapy; one patient, a woman with diffuse calcific plaques determining multiple non-significant stenosis in at least two vessels at CT, underwent conventional coronary angiography and percutaneous revascularization 1 year after CT. Among the 17 patients with evidence of significant CAD at CT, 82% (14/17) had been treated (five with percutaneous revascularization, nine with medical therapy) and three not treated, two of them due to contraindications. All treated patients no longer experienced chest pain, whereas non-treated patients reported persistence of symptoms at follow-up interview. All patients with myocardial bridging at CT had been treated if evaluated by cardiologists and almost never treated if evaluated by general practitioners; 62% of them (13/21) were free of chest pain at follow-up. The majority of the patients with non-cardiovascular disease (27/36) were pain-free at follow-up; the patient with pneumothorax and the patient with pneumonia were treated at the time of diagnosis, while 16 of 25 patients with hiatal hernia received appropriate therapy.

**Table 2:** Association between CT results and patient management in the 94 patients with available follow-up

CT result		Patient management			
		Total, *n* (%)	Cardiologic treatment, *n* (%)	Gastroenterological treatment, *n* (%)	Chest pain persistence, *n* (%)
Nothing		34 (36)	5 (5)	8 (9)	7 (7)
Coronary diseases (CD) only	Significant CAD	12 (13)	9 (10)	6 (6)	2 (2)
	Myocardial bridging	11 (12)	2 (2)	2 (2)	4 (4)
	Malignant coronary anomaly	1 (1)	1 (1)	0 (0)	1 (1)
CD + ECD	Myocardial bridging + hiatal hernia	8 (8)	6 (6)	5 (5)	3 (3)
	Significant CAD + hiatal hernia	3 (3)	3 (3)	3 (3)	0 (0)
	Significant CAD + myocardial bridging + hiatal hernia	2 (2)	2 (2)	1 (1)	1 (1)
	Malignant coronary anomaly + hiatal hernia	1 (1)	1 (1)	1 (1)	1 (1)
Extracardiac diseases (ECD) only	Hiatal hernia	20 (21)	8 (9)	11 (12)	4 (4)
	Pneumothorax*	1 (1)	0 (0)	0 (0)	0 (0)
	Pneumonia* + cholecystitis*	1 (1)	0 (0)	0 (0)	0 (0)
Total		94 (100)	39 (42)	37 (39)	23 (24)

*All these patients had the acute disease (pneumothorax, pneumonia, and cholecystitis) treated at the time of diagnosis

Ten patients had undergone a provocative test during follow-up; eight of them had negative results, one positive result (with subsequent medical treatment), and one positive result (with percutaneous revascularization 1 year after CT).

## Discussion

CTCA has evolved into an effective technique for the evaluation of CAD in selected patients, mainly those at low-to-intermediate cardiovascular risk [9, 10, 16, 17]. In our study, patients at any risk class were potentially included. However, our inclusion criteria (chest pain and absence of known CAD history) have limited the inclusion of patients at either too high or too low CAD risk. As a consequence, the rate of patients with patent coronary arteries or non-significant CAD (77%) was expected [18–20].

We investigated one relevant advantage of CT over other cardiac imaging methods, i.e., the possibility to evaluate not only the heart but also the presence of alternative causes of retrosternal chest pain [10, 16, 17]. Forty-three percent of patients presented coronary diseases (23% significant CAD). Including non-cardiovascular diseases, at least one clinically relevant finding was detected in 65% of patients.

Many authors underlined the high prevalence of extracardiac findings at cardiac CT. Flor et al. [16] stated that cardiac CT shows at least one incidental extracardiac finding in almost one of every two patients. However, in the literature, there is a high heterogeneity in terms of prevalence and clinical relevance of these findings, probably due to differences in definition, classification, and reporting [21–23]. Findings such as hiatal hernias may be considered benign and not reported in the final impression, but are potentially important findings [23]. A better integration with clinical data could sometimes avoid the need to perform CTCA. However, if non-cardiovascular disease is not suspected, CT can represent the trigger for diagnosis and treatment.

The comparison between our results and those of other studies is not easy. Koonce et al. [23] included a large patient population but only analyzed the radiological reports of any kind of cardiac CT (calcium score, CTCA, pulmonary vein evaluation, follow-up of coronary artery bypass graft) [23]. Conversely, our study had a prospective design, was conducted only on patients with retrosternal chest pain without previous CAD history, and focused on findings which could explain the chest pain. The majority of the other studies were retrospective series of patients with or without symptoms and CAD, considering any kind of findings [11, 12, 22–25].

The evaluation of non-cardiac structures is a key point in these patients: chest pain is common and disabling, and often persists after a normal cardiac study. Patient management is often inefficient and associated with a significant economic burden [10, 11]. Onuma et al. found extracardiac findings on CT scans considered to be sufficient to explain the symptoms by providing an alternative diagnosis in about 16% (32/201) of patients in whom CAD was ruled out [10, 24]. In our study, instead, the percentage of patients with extracardiac diseases possibly explaining symptoms was 35%, which is clearly higher. Of note, cardiac CT can be performed with an acceptably low radiation dose and can be useful for symptomatic patients even at low risk of CAD [26, 27].

As expected, we did not find any significant association between the kind of chest pain and disease, making CTCA a problem-solving diagnostic tool [1, 2, 10]. In particular, patient management was immediately changed for one patient with pneumothorax and one patient with cholecystitis and pneumonia, avoiding potential delay in patient care or even misdiagnosis. Also, patients with suspected myocarditis and no coronary stenosis at CTCA (seven patients in our series) were promptly treated [10].

Notably, large discrepancies were found in the literature between the prevalence of clinically relevant findings and the number of findings that actually led to therapeutic or diagnostic interventions [21]. Our patients with CAD evidence at CTCA all underwent a cardiologic evaluation and were treated with percutaneous revascularization or medical therapy (whenever possible), confirming the reliability of CTCA [13, 20]. We noted a difference in the management of patients with myocardial bridging, a benign condition that requires treatment when symptomatic, frequently detected at CT (21% in our population). While most of these patients were treated if evaluated by cardiologists, they were almost never treated if evaluated by general practitioners, probably because this is an unknown condition for non-cardiologists.

Our study has limitations. The first is the relatively small sample size (*n* = 106). However, patient selection was narrowed to only patients experiencing retrosternal chest pain, without previous CAD history. Second, the relationship between chest pain and CT findings (especially hiatus hernias and myocardial bridging) was not always completely clarified by our follow up. As a consequence, the attribute of clinical improvement to the treatment has some degree of uncertainty. In any case, the suspicion of non-cardiovascular disease from CT findings can be a valid aid for clinical decision-making.

In conclusion, our results showed that CTCA suggests possible causes of retrosternal chest pain in 65% of patients, with a relatively high prevalence (22%) of only relevant non-cardiovascular findings, which need to be carefully examined by radiologists.

## Abbreviations

CTCA: Computed tomography coronary angiography
CAD: Coronary artery disease
CD: Coronary diseases
ECD: Extracardiac diseases
BMI: Body mass index
CT: Computed tomography
ECG: Echocardiogram
MPR: Multi-planar reformatted
CPR: Curved planar reformatted
MIP: Maximum intensity projection
VR: Volume rendering
SD: Standard deviation
